# Multifactorial Diseases of the Heart, Kidneys, Lungs, and Liver and Incident Cancer: Epidemiology and Shared Mechanisms

**DOI:** 10.3390/cancers15030729

**Published:** 2023-01-25

**Authors:** Canxia Shi, Sanne de Wit, Emina Učambarlić, George Markousis-Mavrogenis, Elles M. Screever, Wouter C. Meijers, Rudolf A. de Boer, Joseph Pierre Aboumsallem

**Affiliations:** 1 Department of Cardiology, University Medical Center Groningen, University of Groningen, 9700 RB Groningen, The Netherlands; c.shi@umcg.nl (C.S.); s.de.wit@umcg.nl (S.d.W.); emina.ucambarlic@gmail.com (E.U.); g.markousis.mavrogenis@umcg.nl (G.M.-M.); e.screever@erasmusmc.nl (E.M.S.); w.c.f.w.meijers@umcg.nl (W.C.M.); 2 Department of Cardiology, Erasmus MC, 3000 CA Rotterdam, The Netherlands

**Keywords:** incident cancer, onco-nephrology, chronic obstructive pulmonary disease, heart failure, cardio-oncology, reverse cardio-oncology, end-stage renal disease, metabolic associated fatty liver disease

## Abstract

**Simple Summary:**

Multifactorial diseases are caused by a combination of genetic and environmental factors and various risk factors that accumulate with age. Cardiovascular diseases, chronic kidney disease, chronic obstructive pulmonary disease, metabolic (dysfunction) associated fatty liver disease, and cancers are the most common multifactorial diseases and impose a considerable healthcare burden. The simultaneous manifestation of two or more of these diseases represents various clinical challenges as each disease interferes with the treatment of the other. This review summarizes several lines of evidence concerning the bi-directional relationship between multifactorial diseases and cancer. Moreover, this article aims to increase clinicians’ awareness regarding the risk of cancer development among patients with other multifactorial diseases.

**Abstract:**

Within the aging population, the frequency of cancer is increasing dramatically. In addition, multiple genetic and environmental factors lead to common multifactorial diseases, including cardiovascular disease, chronic kidney disease, chronic obstructive pulmonary disease, and metabolic-associated fatty liver disease. In recent years, there has been a growing awareness of the connection between cancer and multifactorial diseases, as well as how one can affect the other, resulting in a vicious cycle. Although the exact mechanistic explanations behind this remain to be fully explored, some progress has been made in uncovering the common pathologic mechanisms. In this review, we focus on the nature of the link between cancer and common multifactorial conditions, as well as specific shared mechanisms, some of which may represent either preventive or therapeutic targets. Rather than organ-specific interactions, we herein focus on the shared mechanisms among the multifactorial diseases, which may explain the increased cancer risk. More research on this subject will highlight the significance of developing new drugs that target multiple systems rather than just one disease.

## 1. Introduction

During the late 20th and early 21st centuries, a pronounced shift in global demographics towards older ages became more and more evident, reflecting the development of public health systems, improvements in the practice of medicine, and amelioration of socio-economic standards. According to the United Nations Department of Economic and Social Affairs Population Division 2019, the worldwide population over 65 is expected to exceed 1.5 billion in 2050 [1]. Age greatly increases the risks of chronic diseases and is associated with a loss of reparative and regenerative capacities in several organs. In addition, there is a decrease in physiological reserve capacities in response to stress and time-dependent, cumulative alterations of critical molecular pathways leading to organ dysfunction [2].

Multifactorial diseases are caused by a combination of genetic and environmental factors, as well as various risk factors that accumulate with age. Cardiovascular diseases (CVD), chronic kidney disease (CKD), chronic obstructive pulmonary disease (COPD), metabolic (dysfunction) associated fatty liver disease (MAFLD), and malignancies are the most common multifactorial diseases, and impose a considerable healthcare burden [3], particularly since their prevalence strongly increases with age [4,5,6,7,8,9,10].

Recently, the bidirectional links between multifactorial diseases and cancer attracted scientific interest. Cancer treatment and management are associated with potential sequelae, including but not limited to cardiotoxicity, pulmonary toxicity, renal injury, and liver damage [11,12,13,14,15]. Consequently, scientific fields such as cardio-oncology and onco-nephrology were established through surveillance and interventions to prevent and reduce the adverse effects of anticancer treatments. But rather than organ-specific interactions, the relation between cancer and multifactorial disease is explained by generic mechanisms, that are shared between these diseases. This review summarizes the increasing evidence pertaining to the link between incident cancer and several common multifactorial diseases including heart failure (HF), renal failure/CKD, COPD, and MAFLD. Moreover, this article serves to inform clinicians regarding the increased risk of cancer development among patients with other multifactorial diseases.

## 2. Heart Failure Triggers a Pro-Oncogenic Milieu

### 2.1. The Bidirectional Relationship between Cancer and Heart Failure

Cardiotoxicity induced by cancer therapy is gradually accumulating and becoming increasingly evident despite an increased survival rate for cancer patients. For instance, anthracycline-based treatment can result in 5–48% irreversible cardiac damage and HF in a dose-dependent manner [16]. Emerging evidence also supports the fact that more than 40% of cancer patients’ death are attributed to cardiovascular disease [17]. Therefore, cardio-oncology emerged as a new field that focuses on the monitoring, detection, and treatment of CVD and the optimization of cancer therapies in cancer patients [12,18,19,20]. Interestingly, recent epidemiologic data have demonstrated that cancer prevalence is higher in patients with HF compared to the general population [21,22]. HF patients have a 24–68% increased risk of developing cancer, and cancer mortality was significantly higher in HF patients compared to healthy subjects [23,24,25]. These observations have been attributed by some to surveillance bias, i.e., malignancies may be detected more frequently due to routine monitoring for HF management. Moreover, HF and cancer have shared risk factors, including hypertension, obesity, diabetes mellitus, smoking, and reduced physical activity [25], which could explain their concurrent manifestation.

We have extensively discussed the bidirectional link between these two syndromes and provided a 5-tier classification system to categorize cardio-oncology syndromes (COS) that characterize the features of the link between cancer and cardiovascular diseases. In summary, COS Type I represents the mechanisms by which cancer can lead to cardiovascular dysfunction. COS Type II comprises the mechanisms by which cancer therapies can result in acute or chronic CVD. COS Type III refers to the pro-oncogenic milieu created by the release of cardiac factors. COS Type IV includes CVD management, including therapies and diagnostic practices that have been associated with promoting or unmasking malignancies. COS Type V refers to common factors causing systemic and genetic predisposition to both diseases [20]. Moreover, a wealth of preclinical and epidemiological analyses lend support to the postulation that HF is a pro-oncogenic condition for incident cancer. We have highlighted the common mechanistic pathways in cancer and heart failure [19] and designed a roadmap with key steps to guide and improve future clinical and pre-clinical research and increase the collaboration between cardiologists and oncologists [19].

### 2.2. Preclinical Data: Heart Failure Accelerates Tumour Growth

The first preclinical study to assess the effect of HF on cancer development discerned the role of myocardial infarction (MI)-induced HF on intestinal polyp formation in the APC^min^ mouse model. The authors found that HF resulted in an increased intestinal tumor load and the severity of HF was strongly associated with tumor growth, independent of hemodynamic changes. This was explained by factors secreted by failing hearts, which stimulated the proliferation of colon cancer cells [24]. This study provided the first key evidence in a preclinical model that HF represents a systematic pro-oncogenic environment that can directly stimulate colon cancer growth. A subsequent basic study revealed that MI accelerates breast cancer outgrowth by epigenetically reprogramming Ly6C^hi^ monocytes to an immunosuppressive phenotype in the circulation and tumor [26]. Compared to the sham group, MI increased the proportion of Ly6C^hi^ monocytes in tumor tissue, enhanced the chromatin accessibility of Ly6C^hi^ monocytes in pathways regulating stress responses, and reduced the chromatin accessibility in pathways related to immune and inflammatory responses. For instance, the binding sites of PU.1, CCAAT-enhancer-binding protein (CEBP), and interferon regulatory factor (IRF)-8 of Ly6C^hi^ monocytes were less accessible after MI, which impaired myeloid cell differentiation and transcriptionally inhibited numerous genes regulated by PU.1, CEBP and IRF-8 such as CD40 and CD86 genes involved in T cell activation, resulting in an immunosuppressive phenotype that persists in tumor monocytic myeloid-derived suppressor cells (mMDSCs) [26]. The authors proposed that MI-induced HF resulted in systemic hematopoiesis and an immunosuppressive milieu that altered the normal phenotype of monocyte precursors, and eventually promoted tumor growth.

Cancer progression was also assessed in another mouse model of a different HF etiology, namely, pressure overload-induced cardiac hypertrophy. The transverse aortic constriction (TAC) model was performed, followed by cancer cell implantation of breast cancer or lung cancer cells. TAC-operated mice displayed larger tumors and more severe metastatic lesions in the lung compared to control groups [27]. Similarly, the authors identified that periostin, an extracellular matrix protein secreted by the remodeled hearts, was elevated in the serum after TAC surgery and was able to stimulate cancer progression in vitro.

### 2.3. Common Risk Factors and Signalling Pathways

**Figure 1:**
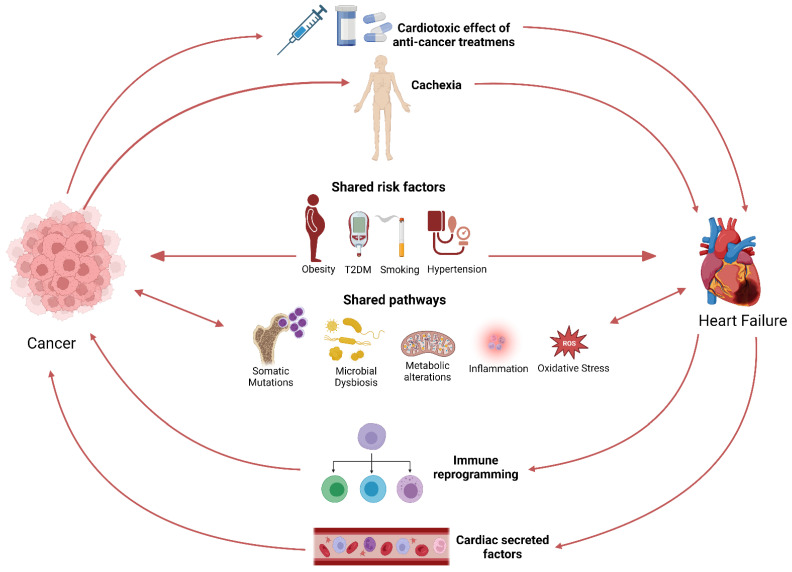
Schematic representation of the bi-directional link between cancer and HF.

Shared pathways underlying the pathogenesis of both HF and cancer (Figure 1), such as inflammation, oxidative stress, and somatic mutations, in part explain the coexistence of these two syndromes [19,28]. Clonal hematopoiesis of indeterminate potential was shown to be associated with incident HF, HF risk factors, and biomarkers [29,30]. In addition, established tumor biomarkers predicted cardiovascular outcomes in HF patients and correlated with cardiovascular events in a general population [31,32]. Another layer of evidence from clinical studies supports the link between HF and cancer. Interestingly, the microbiome has recently emerged as a common pathway and a potential link between the two diseases [33]. Gut microbial dysbiosis is associated with several types of cancer, including colorectal cancer (CRC), liver cancer, and pancreatic cancer [34,35,36]. In addition, microbial dysbiosis has been observed in patients with HF [37,38]. Recent studies have provided evidence that pathogenic bacteria can possess tumorigenic effects [34]. In addition, pathogenic bacteria can induce chronic inflammation, which is associated with CVD [39]. Several microbial metabolites, such as TMAO, have also been associated with an increased risk for CVD and cancer [40,41,42].

Nowadays, cardiologists and oncologists recognize the cardiotoxic effects of antineoplastic treatments, which has resulted in the establishment of cardio-oncologic clinics. Nevertheless, less awareness is given to ‘’reverse cardio-oncology’’, where HF itself constitutes a risk factor for cancer. Exploring pathways theoretically linking HF to cancer is a growing field of research, with the goals of understanding the bidirectional link between HF and cancer, finding common therapeutic targets, and improving the treatment of patients.

## 3. Kidney Disease: A Potential Risk Factor for Cancer

### 3.1. Cancer Risk in Chronic Kidney Disease Patients

Throughout the last two decades, the link between CKD and other diseases including cancer has progressively become acknowledged. CKD and malignancy are correlated in several ways in both directions. [43] It is well known that nephrotoxicity and CKD might be caused by anti-neoplastic therapies and occur after nephrectomy in patients with kidney cancer [44,45,46]. Conversely, CKD may also lead to cancer via an underlying cystic disease, increased urinary concentrations of carcinogenic toxins, oxidative stress, and inflammatory milieu [47,48].

It remains unclear at which stage of CKD cancer incidence starts to rise. A cohort study reported a higher cancer risk in patients with moderate CKD, although this trend was only observed in men [49]. Cancer prevalence increased from an estimated glomerular filtration rate (eGFR) of 55 mL/min/1.73 m^2^ and kept rising linearly as eGFR was decreasing. Independent of smoking and age, the authors found that each 10 mL/min/1.73 m^2^ reduction in eGFR was associated with a 29% increased risk of new-onset cancer. This association appeared to be cancer site specific, and was more significant in lung and urinary tract malignancies [49]. A retrospective cohort study also demonstrated that reduced eGFR correlated with elevated risks of renal and urothelial cancer [50]. Comparable trends were observed in another cohort with a younger and larger population. The investigators found a positive correlation between overall cancer incidence and reduced kidney function (log of albumin to creatinine ratio), which is mainly exposed in lung, colon, kidney, and bladder cancers [51]. However, a meta-analysis of six prospective studies did not show a significant association between reduced kidney function and overall cancer incidence, but among dialysis patients, the risk of cancer in the urinary tract, endocrine, and digestive tract was significantly increased [52]. Therefore, the link between CKD and cancer incidence should be evaluated with a clear stratification of cancer sites and certain CKD categories.

Cancer incidence for up to five years has been evaluated among patients with end-stage renal disease (ESRD) and before they underwent renal replacement therapy. These patients demonstrated a higher risk of several cancer types such as renal cell carcinoma, bladder cancer, multiple myeloma, sarcoma, and lymphoma [53]. Correspondingly, an international collaborative study gathered a cohort of 831,804 CKD patients who received dialysis [54]. The investigators found a higher cancer prevalence in the kidney, bladder, and thyroid. Several studies addressed cancer risk in patients during dialysis and with renal transplantations and are presented in Table 1 and Table 2, respectively.

### 3.2. Mechanisms Linking Kidney Disease to Cancer Development

One of the main features of CKD is a persistent inflammatory state associated with high production of pro-inflammatory cytokines, such as interleukin-1β (IL-1β), interleukin-6 (IL-6), and tumor necrosis factor α (TNF-α). It is well established that inflammation is one of the main risk factors and a key mechanism in cancer formation and development. An inflammatory milieu allows malignant cells to escape host immune surveillance, stimulating angiogenesis, tumor growth, and invasiveness. An incessant low and chronic inflammation in CKD patients will activate the generation of nicotinamide adenine dinucleotide phosphate (NADPH) oxidase and myeloperoxidase (MPO) by polymorphonuclear neutrophils and monocytes macrophages, which promotes the production of reactive oxygen species (ROS) and initiates a state of oxidative stress [62]. Under pro-oxidative stress conditions, ROS are continually produced by aerobic metabolism in the mitochondria, which results in severe damage to cell structure and function and induces somatic mutations and tumor formation [63]. The latter process involves increased DNA mutations, DNA damage, genomic changes, and cancerous cell proliferation [64].

**Figure 2:**
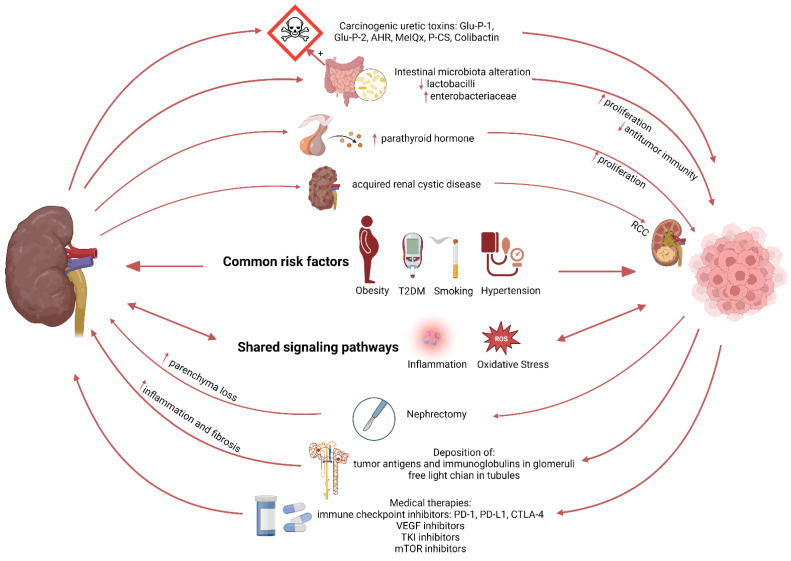
Schematic representation illustrating the relation between kidney disease and cancer.

Patients with ESRD have higher levels of carcinogenic compounds and nitrogen-containing substances accumulating in the blood [65]. Carcinogenic compounds such as 2-amino-6-methyldipyrido [1,2-a: 3′,2′-d] imidazole (Glu-P-1) and 2-aminodipyrido [1,2-a:3′,2′- d] imidazole (Glu-P-2) are relatively higher in plasma of uremic patients undergoing dialysis compared to the healthy population, and potentially bind to DNA and cause DNA damage [66,67]. In addition, uremia can affect the composition of the intestinal microbiota and intestinal barrier, and further promote pathogen overgrowth, and bacterial translocation from the gut into mesenteric lymph nodes, liver, and spleen [66]. The increased bacterial translocation will activate innate immunity and systemic inflammation, and the imbalance of intestinal flora can increase the production of toxins such as Colibactin to induce DNA damage and tumor-promoting metabolites such as secondary bile acids [48]. More identified bidirectional links between CKD and cancer pathogenesis are illustrated in Figure 2.

## 4. Lung Cancer Development in Chronic Obstructive Pulmonary Disease

COPD and lung cancer are two leading public health issues and causes of morbidity and mortality. Both diseases share common risk factors such as exposure to smoking and genetic predisposition. Approximately 15–20% of lifelong smokers will develop COPD or lung cancer [68]. Furthermore, COPD is highly associated with up to a 4.5-fold increased risk of lung cancer and represents a major independent risk factor for lung cancer among smokers [69]. Even within mild COPD, emphysema has already been shown to be associated with the occurrence of lung cancer [70]. The major pathophysiologic drivers in this context include genetic predisposition, oxidative stress, inflammation, and inflammatory mediators [71].

### 4.1. Shared Susceptibility Loci and DNA Epigenetic Modification in COPD and Lung Cancer

Some well-known gene families of proteinases, detoxifying enzymes, and inflammatory cytokines play roles in COPD development [72]. Several genome-wide association studies (GWAS) also identified that certain chromosomal regions and candidate genes such as glycophorin A (GYPA), hedgehog interacting protein (HHIP), and family with sequence similarity 13 member A (FAM13A) were associated with the susceptibility to both COPD and lung cancer [72,73,74,75,76]. Interestingly, single nucleotide polymorphisms (SNPs) in the nicotinic acetylcholine receptor (nAChRs) subunit genes, CHRNA3 and CHRNA5, mapped at chromosome 15q25, have been linked to the increased risks of both COPD and lung cancer [76,77,78]. These findings suggest that shared pathogenetic pathways may underlie susceptibility to these two smoking-related diseases. 

Epigenetic modification is another important link between COPD and lung cancer [79]. Common methylation marks and changes in gene expression, most probably induced by smoking, are observed in both patients with lung cancer and COPD [80]. DNA hypermethylation has been identified as an important factor in the development of lung cancer, leading to changes in the expression of numerous oncogenes and tumor suppressor genes [81,82]. Two of them, cyclin-dependent kinase inhibitor 2A (CDKN2A), which encodes the tumor suppressor p16, and O-6-methylguanine-DNA methyltransferase (MGMT), are methylation targets shared by COPD and lung cancer [83,84]. A recent genome-wide epigenetic study discovered 349 CpG sites that were strongly associated with COPD [85]. Interestingly, many of these sites have previously been associated with an increased risk of lung cancer. Furthermore, a bioinformatic analysis of DNA methylation patterns in COPD-associated lung cancer revealed dysregulation of innate immunity and lymphocyte trafficking [86]. This supports the view that COPD’s inflammatory environment influences lung cancer by disruption of epigenetic modifications.

### 4.2. Inflammation and Oxidative Stress: Two Shared Signalling Pathways

COPD is characterized by chronic lung inflammation as well as immune cell recruitment and activation [87]. Inflammation as a link between COPD and lung cancer has been suggested, but the exact underlying mechanism is unknown. Inflammatory mediators released into bronchial epithelial stem cells promote inflammation-induced cancer by causing cellular proliferation, resistance to apoptosis, invasion, and metastasis [88,89]. For example, the inflammatory cytokine TNF-α is upregulated in COPD patients and is known to promote tumor cell proliferation and differentiation [90,91]. Furthermore, matrix-metalloproteinases (MMPs), particularly MMP-9, and MMP-2, are overexpressed in COPD patients and are involved in tissue remodeling, emphysema development, cancer cell proliferation, invasion, and metastasis [92,93,94,95]. Interestingly, MMP-12, another highly expressed protease in COPD, whose activity has been linked to disease severity, is known to be tumor suppressive and thus not optimal as a target for the treatment of cancer [96].

**Figure 3:**
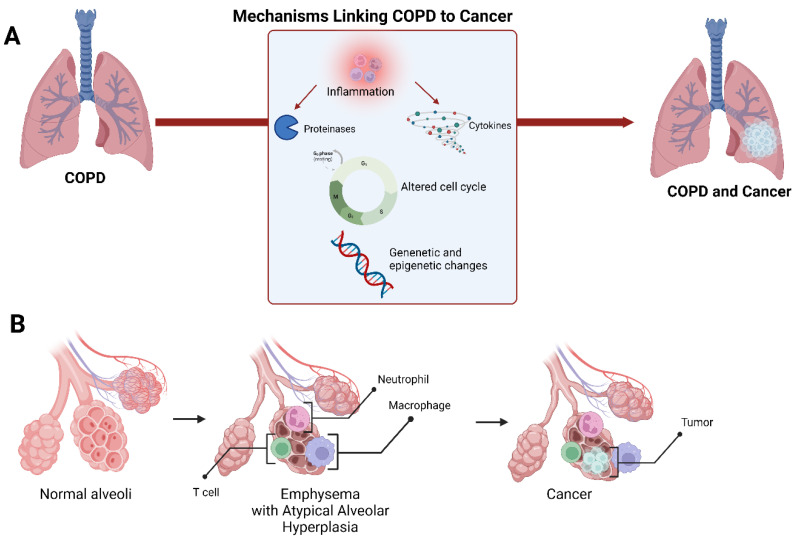
Schematic representation of the mechanisms linking COPD to lung cancer (**A**). A suggested model of lung cancer development in the setting of emphysema at the alveolar level (**B**).

Another major driving mechanism in the pathogenesis of COPD is oxidative stress, which is well established as being causative for cellular proliferation in lung cancer [97]. The dysfunction of mitochondria in the airways and lung parenchyma can influence COPD pathogenesis, including an imbalance of oxidative stress, which can amplify chronic inflammation and promote carcinogenesis (Figure 3) [98].

The NF-kappa-B transcription complex (NF-κB) molecule is an important transcription factor that promotes the production of inflammatory mediators [99]. The NF-κB pathway was found to be persistently activated in the airway epithelium of COPD patients as well as in neoplastic cells of squamous cell cancers [100]. Increased activation of this pathway can lead to emphysema, small airway remodeling, and, finally, accelerate cancer development [101]. Phosphorylation and acetylation regulate the activity of NF-κB -p65, a subunit of NF-κB. Sirtuin-1 (SIRT1), a protein deacetylase, has been shown to deacetylate NF-κB-p65 and suppress stimuli-induced NF-κB activation [102]. This protein, however, is reduced in the lungs of COPD patients [103]. The Akt/mTOR pathway, which is negatively regulated by the SIRT1 protein, is upregulated in lung cancer patients with mild COPD [104]. Signal transducer and activator of transcription 3 (STAT3) is another interesting transcription factor that plays a role in many biological functions. Increased activation of the STAT3 signaling pathway contributes significantly to lung inflammation and adenocarcinoma formation [105].

Although the exact mechanism underlying COPD and lung cancer is not fully explored, growing evidence suggests that the two diseases may be linked at the molecular level. However, the available literature is limited to animal models and small clinical trials. Large clinical trials and combined models of COPD and lung cancer are required to investigate the processes that link COPD and lung cancer.

## 5. The Progression of Metabolic Associated Fatty Liver Disease to (Extra)-Hepatic Cancers

### 5.1. Metabolic (Dysfunction) Associated Fatty Liver Disease

MAFLD, previously known as non-alcoholic fatty liver disease (NAFLD), is the most common cause of chronic liver disease in Western countries. The change in nomenclature to MAFLD was recently proposed by a group of experts to better emphasize the essential role of metabolic disorders in the pathogenesis of fatty liver disease [106]. MAFLD is diagnosed based on the evidence of hepatic fat accumulation, using biopsy or biomarkers, in combination with one of the following criteria: overweight/obesity, T2DM, or evidence of at least two metabolic abnormalities [106].

Metabolic disorders are the major causes of MAFLD. Excessive fat intake, as consumed with the western diet, leads to lipid uptake in the liver far extending the ability to oxidize the lipids or to export via very low-density lipoprotein (VLDL), resulting in hepatic lipid accumulation [107]. In addition, the high density of simple carbohydrates, such as fructose, in the Western diet can attribute to the development of MAFLD. Fructose is mainly metabolized in the liver to triglycerides via de novo lipogenesis, which results in an increased hepatic fat content [108]. Insulin resistance and high glucose intake also aggravate MAFLD development, by stimulating de novo lipogenesis resulting in increased ROS production [109].

### 5.2. MAFLD: A Multisystem Disease

**Figure 4:**
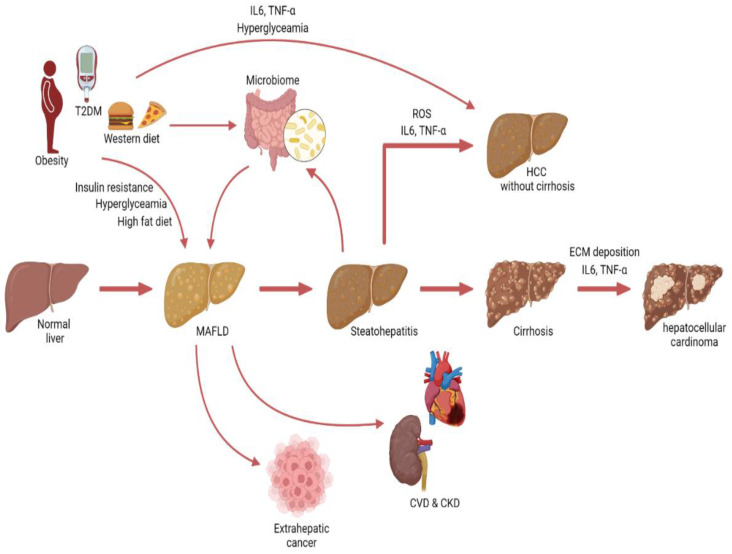
Graphical illustration depicting the progression from MAFLD to hepatocellular carcinoma and non-hepatic conditions.

MAFLD may be considered a multisystem disease [110,111] (Figure 4). MAFLD has been associated with an increased risk for cancer [112], as well as CVD and CKD [113,114]. Patients with MAFLD are at increased risk for major adverse cardiovascular events and CVD mortality [115,116]. In addition, MAFLD was shown the be a predictor of CKD risk [114]. However, some studies showed that MAFLD was not independently associated with CKD risk, but that the link between MAFLD and CKD is mainly driven by the underlying metabolic abnormalities [117].

The association between MAFLD and hepatocellular carcinoma (HCC) has been studied most extensively. MALFD significantly increases the risk for HCC [115,118,119]. In addition, the presence of MAFLD in patients with chronic hepatitis B increased the risk for HCC [120,121,122]. However, multiple studies showed that the metabolic disorders in MAFLD patients were the most important contributors to the increased cancer risk [115,123,124].

MAFLD has not only been associated with an increased risk for HCC but also with several extrahepatic cancers, including CRC, kidney cancer, thyroid cancer, and breast cancer [110,111,112,119]. Interestingly, the risk for kidney and thyroid cancer remained significant even after adjustment for waist circumference and metabolic syndrome [112]. The association between MAFLD and CRC has been gathering increasing attention over the last few years. Several epidemiological studies showed that MAFLD is an independent risk factor for CRC, even after adjustments for age, metabolic syndrome, and diabetes [95,125,126,127,128].

### 5.3. Mechanisms Linking MAFLD to HCC

Over the last few years, MAFLD has become the fastest-rising cause of HCC in the US [129]. Obesity and T2DM play an important role in HCC development and progression in MAFLD [130]. It is well established that obesity increases the risk of cancer in general. The chronic low-grade inflammation from the adipose tissue, triggers secretion of IL-6 and TNF-α, which are both associated with tumorigenesis [131,132]. In addition, diabetes can affect carcinogenesis via several mechanisms, including hyperinsulinemia, hyperglycemia, and chronic inflammation [133].

However, fatty liver disease itself can also affect HCC. Cirrhosis is a major risk factor for HCC. Epidemiological studies indicate that in 11–38% of the HCC patients cirrhosis was identified as the underlying etiology [125,126]. In cirrhosis, stellate cells are activated by inflammatory and pro-fibrotic signals from hepatocytes and immune cells, leading to increased extracellular matrix deposition, instigating a favorable microenvironment for tumor cells [127,128].

Accumulating evidence suggests that steatohepatitis can also stimulate tumorigenesis, irrespective of cirrhosis, in several ways. Recent studies showed that in individuals with steatohepatitis, 12–14% of cancers occur prior to cirrhosis [134,135,136]. Steatosis can lead to mitochondrial dysfunction and endoplasmic reticulum (ER) stress, resulting in increased ROS production [137,138]. Oxidative stress is known to lead to DNA damage and activation of oncogenic pathways such as NF-κB [139]. Steatosis also leads to impaired autophagy, which is important for catabolizing lipids. Impaired autophagy leads to ER stress, resulting in increased ROS and inflammation [140,141]. The chronic inflammatory state in people with steatohepatitis also contributes to hepatic carcinogenesis. Steatohepatitis is characterized by an increase in proinflammatory cytokines including IL-6 and TNF-α. TNF-α has been studied extensively in cancer research and is known to activate several oncogenic pathways such as the NF-κB and mTOR pathway [142,143]. IL-6 is the major activator of STAT3, which is often increased in steatohepatitis patients, and can induce proliferation and malignant transformation [144]. Inhibition of IL-6 in mice on a high-fat diet was shown to protect against the tumor-promoting effects of the high-fat diet [145].

Interestingly, MAFLD-associated HCC may also be induced by changes in the gut microbiome. Several studies have observed microbial dysbiosis in MAFLD patients [146]. Fatty liver disease is associated with gut barrier dysfunction and increased translocation of bacteria and lipopolysaccharides, which can induce hepatic inflammation and fibrosis [147,148]. In addition, the microbiome regulates the farnesoid X receptor (FXR), which has been shown to have anticarcinogenic effects on the liver [149]. However, it is important to keep in mind that obesity and unhealthy dietary habits affect both MAFLD and microbial composition and should be considered confounders (Figure 4).

These data build on the evidence that MAFLD may be considered a multisystem disease, and with the increasing incidence of MAFLD, it is important to study the connection between MAFLD and other diseases such as cancer (Figure 4).

## 6. Clinical Considerations

In this review, we discuss several lines of evidence suggesting a common pathophysiologic background and a bi-directional relationship between different common multifactorial diseases and cancer. Although the corresponding evidence base has gained increasing scientific interest in recent years, there are still no specific therapeutic interventions with potential clinical applications in this context. Coincidentally, one such example was seen in the landmark CANTOS trial, in which patients with a previous myocardial infarction were treated with canakinumab, a monoclonal antibody targeting IL-1β. It has been shown that canakinumab not only improved cardiovascular outcomes but also reduced the incidence of lung cancer and mortality [150,151]. Although subsequent large phase III trials specifically in lung cancer failed to show benefit from canakinumab [152], it should be noted that patients with known cancers were excluded from recruitment in CANTOS, thus still leaving the question open of whether IL-1β blockade could still prove beneficial in preventing cancer mortality in patients with existing CVD. As such, concerted research efforts are necessary in order to elucidate the exact role of IL-1β as well as other potential therapeutic targets with dual actions on both ends of the bidirectional relationship between cancer and the aforementioned multifactorial diseases.

Inflammation is often a connection point between cancer and the discussed diseases, so it represents one of the central therapeutic target candidates. COPD patients, for example, can be screened for changes in the epidermal growth factor receptor (EGFR) or vascular endothelial growth factor (VEGF) and, if necessary, treated with immunosuppressive drugs. Known ones are EGFR inhibitors such as erlotinib [153] or VEGF-binding monoclonal antibodies such as ramucirumab [154]. Because the presence of receptor mutation determines drug efficacy, specific sub-phenotypes of COPD patients will be susceptible to anti-inflammatory or other treatment(s) that will influence the incidence of lung cancer. In other words, it is critical to combine selective biomarkers, carefully stratify at-risk patients for optimal therapeutic effect, and use specifically targeted therapies to reduce the risk of the patients developing any cancer. In addition, a number of future developments may also accelerate drug discovery in a broader range of chronic inflammatory diseases. Human genetic studies that link specific inflammatory genes to a common mechanism in cancer and other diseases may aid in the identification of useful drug targets. Furthermore, given the significance of oxidative stress in cancer and the diseases discussed here, it has been proposed that antioxidant therapy may be useful in patients’ treatment by reducing inflammation and cancer incidence [155,156]. However, when considering antioxidant therapies, it is important to determine the extent to which oxidative stress plays a role in the pathology. If oxidative stress is a secondary cause of disease rather than the primary one, preventing its generation may have little effect on disease progression. A different or additional therapeutic approach should be considered in that case.

Physicians managing patients with chronic conditions, such as COPD, HF, CKD, or MAFLD, should maintain an increased level of suspicion for the potential pro-oncogenic role of these diseases. This review focuses on common multifactorial diseases that have been shown, in clinical and pre-clinical studies, to have a link with cancer. Nevertheless, it is worth mentioning that there are other common diseases such as prostatic hyperplasia. The latter may share similar signaling pathways with prostate cancer since treatments for benign prostatic hyperplasia may also benefit prostate cancer [157].

**Figure 5:**
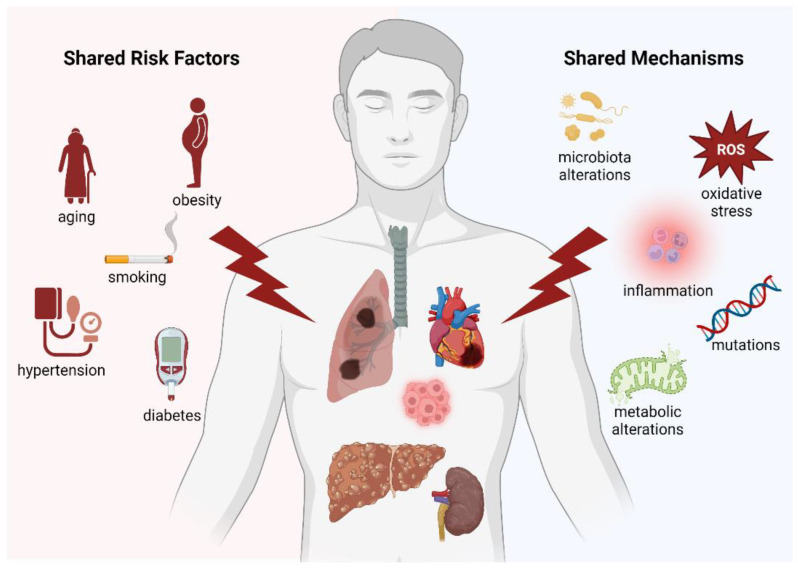
Graphical representation summarizing all the common risk factors/mechanisms between the main multifactorial diseases.

Multifactorial diseases and cancer are all driven by shared pathophysiological pathways (Figure 5), including inflammation, oxidative stress, and mutations. It is also pertinent to consider that a considerable part of the shared pathophysiology between cancer and multifactorial diseases is constituted by preventable or modifiable risk factors, such as smoking, diabetes mellitus, hypertension, hyperlipidemia, obesity, and metabolic syndrome. Thus, it is important to impress upon physicians the fact that these traditionally “cardiovascular“ risk factors may also promote other multifactorial diseases as well as incident cancer [158]. As such, patient education programs and preventive efforts could be modified with this realization in mind. Future research and specifically designed clinical trials based on these crucial observations are currently highly needed and could potentially have a relevant clinical impact on the general health of the increasingly aging world population.

## 7. Conclusions

There is a bidirectional relationship between cancer and common multifactorial diseases (HF, CKD, COPD, MAFLD), and they share common risk factors and a pathophysiologic basis. Although the exact nature of these commonalities and bidirectional interactions remains incompletely understood, a high index of suspicion for incident cancer combined with intensification of patient education and modification of shared risk factors are important supplementary measures that should be followed. Lastly, although there are currently no dual-role therapeutic interventions effective for either end of this bidirectional relationship, potential future targets may include immunomodulatory treatments, modulation of microbiota, and reduction of oxidative stress.

## Figures and Tables

**Table 1:** Risks of cancer in CKD patients during dialysis.

Cancer	Study	Number of Participants	Follow-Up (Years)	Age (Years)	Estimated Risk (95% CI)
Overall	Australia and New Zealand Dialysis and Transplant Registry (ANZDATA) [55]	23,764	mean 2.7	57.5 (43.5–67.6)	SIR 1.45 (1.36–1.54)
Pancreatic	A multicenter retrospective cohort study [56]	6254	mean 2.4	64.0 ± 13.0	SIR 1.17 (0.31–2.99)
Hepatocellular	National Health Institutes Research Database [57]	92,348	mean 4.4	60.4 ± 14.8	SIR 1.4 (1.2–1.5)
Colorectal	A multicenter retrospective cohort study [56]	6254	mean 2.4	64.0 ± 13.0	SIR 1.53 (1.11–2.05)
Bladder	a retrospective cohort including United States (USRDS), Europe (EDTA), Australia, and New Zealand (ANZDATA) [58]	831,804	mean 2.46	mean 55.5	SIR 1.5 (1.4–1.6)
Kidney	ANZDATA [55]	23,764	mean 2.7	57.5 (43.5–67.6)	SIR 5.4 (4.3–6.7)
Lung	National Health Institutes Research Database [57]	92,348	mean 4.4	60.4 ± 14.8	SIR 0.5 (0.5–0.6)
Gastric	A multicenter retrospective cohort study [56]	6254	mean 2.4	64.0 ± 13.0	SIR 1.10 (0.47–2.17)
Thyroid	A multicenter retrospective cohort study [56]	6254	mean 2.4	64.0 ± 13.0	SIR 3.42 (1.25–7.46)
Breast	Meta-analysis including 6 studies [52]	32,057	4.4 (3.2–5.4) for dialysis patients	60 ± 12 for dialysis patients	HR 1.03 (0.50–2.12)
Prostate	Meta-analysis including 6 studies [52]	32,057	4.4 (3.2–5.4) for dialysis patients	60 ± 12 for dialysis patients	HR 0.38 (0.19–0.77)
Leukaemia	National Health Institutes Research Database [57]	92,348	mean 4.4	60.4 ± 14.8	SIR 0.4 (0.2–0.7)
Myeloma	A multicenter retrospective cohort study [56]	6254	mean 2.4	64.0 ± 13.0	SIR 1.31 (0.15–4.72)

Data are presented as values, means ± standard deviation, or medians with interquartile ranges unless otherwise indicated. HR, hazard ratio; SIR, standardized incidence ratio.

**Table 2:** Cancer risks in CKD patients with renal transplantation.

Cancer	Study	Number of Patients with RRT	Follow-Up (Years)	Age (Years)	Estimated Risk (95% CI)
Overall	Australia and New Zealand Dialysis and Transplant Registry (ANZDATA) [55]	8173	mean 6.0	43.4 (31.1–53.9)	SIR 3.03 (2.82–3.25)
Pancreatic	Hong Kong Renal Registry [59]	4674	8.2 ± 6.2	43.7 ± 12.6	SIR 1.57 (0.51–4.87)
Hepatocellular	National Health Insurance Database in Taiwan [60]	4716	4.8 ± 3.1	44.1 ± 12.4	SIR 5.07 (3.89–6.42)
Colorectal	Meta-analysis including 54 studies [61]	1,208,767	NA	NA	SIR 1.40 (1.15–1.71)
Gallbladder	National Health Insurance Database in Taiwan [60]	4716	4.8 ± 3.1	44.1 ± 12.4	SIR 3.02 (0.76–11.99)
Bladder	ANZDATA [55]	8173	mean 6.0	43.4 (31.1–53.9)	SIR 2.6 (1.5–4.2)
Kidney	ANZDATA [55]	8173	mean 6.0	43.4 (31.1–53.9)	SIR 5.0 (3.4–7.1)
Lung cancer	Hong Kong Renal Registry [59]	4674	8.2 ± 6.2	43.7 ± 12.6	SIR 1.68 (1.17–2.42)
Gastric	Hong Kong Renal Registry [59]	4674	8.2 ± 6.2	43.7 ± 12.6	SIR 2.85 (1.62–5.02)
Thyroid	ANZDATA [55]	8173	mean 6.0	43.4 (31.1–53.9)	SIR 3.5 (1.7–6.4)
Breast	Hong Kong Renal Registry [59]	4674	8.2 ± 6.2	43.7 ± 12.6	SIR 1.66 (1–2.75)
Ovarian	Hong Kong Renal Registry [59]	4674	8.2 ± 6.2	43.7 ± 12.6	SIR 3.29 (1.37–7.9)
Leukaemia	Hong Kong Renal Registry [59]	4674	8.2 ± 6.2	43.7 ± 12.6	SIR 2.15 (0.89–5.15)
Myeloma	ANZDATA [55]	8173	mean 6.0	43.4 (31.1–53.9)	SIR 1.8 (0.6–4.2)

Data are presented as values, means ± standard deviation, or medians with interquartile ranges unless otherwise indicated. RRT, renal replacement therapy; SIR, standardized incidence ratio; NA, not available.

## Abbreviations

CDKN2A	Cyclin-dependent kinase inhibitor 2A
CEBP	CCAAT enhancer binding protein
CHRNA	Cholinergic receptor nicotinic alpha
CKD	Chronic kidney disease
COPD	Chronic obstructive pulmonary disease
COS	Cardio-oncology syndromes
CRC	Colorectal cancer
CVD	Cardiovascular disease
eGFR	Estimated glomerular filtration rate
EGFR	Epidermal growth factor receptor
ER	Endoplasmic reticulum
ESRD	End-stage renal disease
FAM13A	Family with sequence similarity 13, member A
FXR	Farnesoid X receptor
Glu-P-1	2-amino-6-methyldipyrido [1,2-a: 3′,2′-d]imidazole
Glu-P-2	2-aminodipyrido [1,2-a:3′,2′- d]imidazole
GWAS	Genome-wide association studies
GYPA	Glycophorin A
HCC	Hepatocellular carcinoma
HF	Heart failure
HHIP	Hedgehog interacting protein
HR	Hazard ratio
IRF	Interferon regulatory factor
IL-1β	Interleukin-1β
IL-6	Interleukin-6
MAFLD	Metabolic (dysfunction)-associated fatty liver disease
MGMT	O-6-methylguanine-DNA methyltransferase
MI	Myocardial infarction
mMDSCs	Monocytic myeloid-derived suppressor cells
MMP	Matrix metalloproteinases
MPO	Myeloperoxidase
NA	Not available
nAChRs	Nicotinic acetylcholine receptors
NADPH	Nicotinamide adenine dinucleotide phosphate
NAFLD	Non-alcoholic fatty liver disease
NASH	Non-alcoholic steatohepatitis
NF-κB	NF-kappa-B transcription complex
RIT	Renal replacement therapy
ROS	Reactive oxygen species
SIR	Standardized incidence ratio
SIRT1	Sirtuin-1
SNPs	Single nucleotide polymorphisms
STAT3	Signal transducer and activator of transcription 3
TAC	Transverse aortic constriction
T2DM	Type 2 diabetes mellitus
TNF-α	Tumour necrosis factor α
US	United states
VEGF	Vascular endothelial growth factor
VLDL	Very low-density lipoprotein
